# Assessment of Patient-Centered Outcomes When Treating Maxillary Constriction Using a Slow Removable Versus a Rapid Fixed Expansion Appliance in the Adolescence Period: A Randomized Controlled Trial

**DOI:** 10.7759/cureus.22793

**Published:** 2022-03-03

**Authors:** Nancy Rabah, Heba M Al-Ibrahim, Mohammad Y Hajeer, Mowaffak A Ajaj, Ghiath Mahmoud

**Affiliations:** 1 Department of Orthodontics, University of Damascus Faculty of Dentistry, Damascus, SYR

**Keywords:** visual analog scale, patient-reported outcome measures, swallowing, mastication, discomfort, pain, slow maxillary expansion, rapid maxillary expansion, posterior crossbite, maxillary constriction

## Abstract

Objective

This study aimed to evaluate and compare the levels of pain, discomfort, and functional impairments between slow and rapid maxillary expansion (RME) in treating skeletal maxillary constriction in the adolescence period (i.e., between 12 and 16 years).

Materials and methods

The study sample consisted of 52 patients (21 males and 31 females) with maxillary skeletal constriction in the posterior region. The patients were randomly distributed into either RME (26 patients, with a mean age of 13.87 (± 1.31) years) or slow maxillary expansion group (SME, 26 patients, with a mean age of 14.31 (± 1.19) years). The levels of pain, discomfort, and functional difficulties were assessed after 24 hours (T1), 7 days (T2), 15 days (T3), one month (T4), and four months (T5) following the onset of the expansion procedure.

Results

Patients in the RME group encountered significantly greater levels of pain and discomfort than those in the SME group at T1, T2, and T3 (p>0.001). Chewing and swallowing difficulties were significantly greater in the RME group at T1, T2, T3, and T4 (P≤0.001). The pressure on soft tissue was greater in the RME group at T2 and T3 (p>0.001). After four months (T5), the levels of pain and discomfort decreased to their lowest levels, as well as the difficulties of chewing and swallowing, and the pressure on soft tissue were almost non-existent in both groups.

Conclusion

Patients treated with the removable slow maxillary expander reported lower levels of pain and discomfort, fewer chewing and swallowing difficulties, and less pressure on soft tissues than those treated with the bonded rapid maxillary expander. These difficulties gradually decreased over time in both groups. The lower levels of pain and discomfort may make the SME an effective and comfortable treatment alternative for adolescents with skeletal maxillary constriction.

## Introduction

Skeletal maxillary constriction is a prevalent orthodontic malocclusion that can be seen at any age [1]. The maxillary expansion is the most significant treatment choice for this skeletal problem in the upper jaw [2]. It depends on the ability to open the palatal suture at young ages [1,3]. There are numerous types of maxillary expansion in the mixed dentition and permanent dentition in terms of the applied force and the frequency of expansion activations: slow maxillary expansion (SME), rapid maxillary expansion (RME), and semi-rapid maxillary expansion (SRME) [1,2,4]. However, in adult patients when the ability to expand the maxilla through suture opening is almost absent, surgically assisted rapid maxillary expansion is used as an alternative option [5].

Practitioners are aware that complaints reported by children and adolescents during the active phase of expansion, such as pain, discomfort, and irritations, are common symptoms [6]. The latest studies have shown that the SME has been similar to the RME in its dentoalveolar effects and its effects on the midpalatal suture [3]. All side effects caused by orthodontic appliances have a negative effect on the acceptance of these appliances, which impacts patients' cooperation [7]. It is known that the success of treatment with orthodontic appliances, whether they are fixed or removable, depends on patient cooperation, whatever the appliance type used [8,9].

In the previous literature, a large number of trials have studied pain and discomfort accompanying different orthodontic procedures, such as elastomeric separation [10], mini implant insertion [7], archwire sequences [11,12], removable appliances [13], functional appliances [14], and intermaxillary elastics [7]. Some studies have investigated the pain and discomfort associated with rapid maxillary expansion [15-20]. A few of these studies have compared the different modalities of rapid maxillary expansion in terms of pain and discomfort [17-19]. It has been shown that the majority of RME patients have experienced different levels of pain [15,18,20]. Ugolini et al. found that pain starts during the initial phase of activation [21]. The pain increased to reach its highest levels in the first four days, with a maximum peak on the second day, then gradually diminished [21]. Needleman et al. reported that the greatest pressure generated by every activation existed instantly after turning the jackscrew, with the highest intensity of pain during the first six turns [20]. However, it has been shown that the intensity of pain reported during RME can be influenced by the activation protocol, the daily number of activations, and skeletal maturity [18].

After critically examining the current literature, it seems that there is no trial that has compared the removable slow maxillary expansion with the bonded rapid maxillary expansion in terms of pain, discomfort, and functional impairment. Therefore, this study aimed to evaluate patient-reported outcomes during the rapid maxillary expansion using a modified Hyrax-type RME with an acrylic bonded cover compared to the slow maxillary expansion using a removable expansion plate in the adolescence period (i.e., between 12 and 16 years).

## Materials and methods

Study design and registration

This randomized single-center controlled trial with two parallel groups was designed to compare the pain and discomfort accompanying the rapid and slow maxillary expansion in adolescent patients. Registered patients at the Department of Orthodontics at Damascus University, Dental School, were examined between September 2020 and January 2021. The Local Research Ethics Committee Approval of the University of Damascus was obtained (UDDS-576-2020DG/SRC-5389). This study was registered at ClinicalTrials.gov (ID: NCT05248087) retrospectively. It was funded by Damascus University, Postgraduate Research Budget (Ref no: 66734567781CVN).

Sample size calculation

The sample size was determined using Minitab® Version 17 (Minitab Inc., State College, Pennsylvania, USA). It was assumed that the smallest detectable difference in pain levels between the two groups was 1 cm on a VAS (with a standard deviation of 1.2 from a previous study) [21]. Using an independent-samples t-test with a significance level of 5% and a power of 80%, 24 patients were required in each group. Two patients were added to each group to avoid any potential attrition, bringing the total number of patients in each group to 26.

Patients' recruitment and eligibility criteria

**Figure 1:**
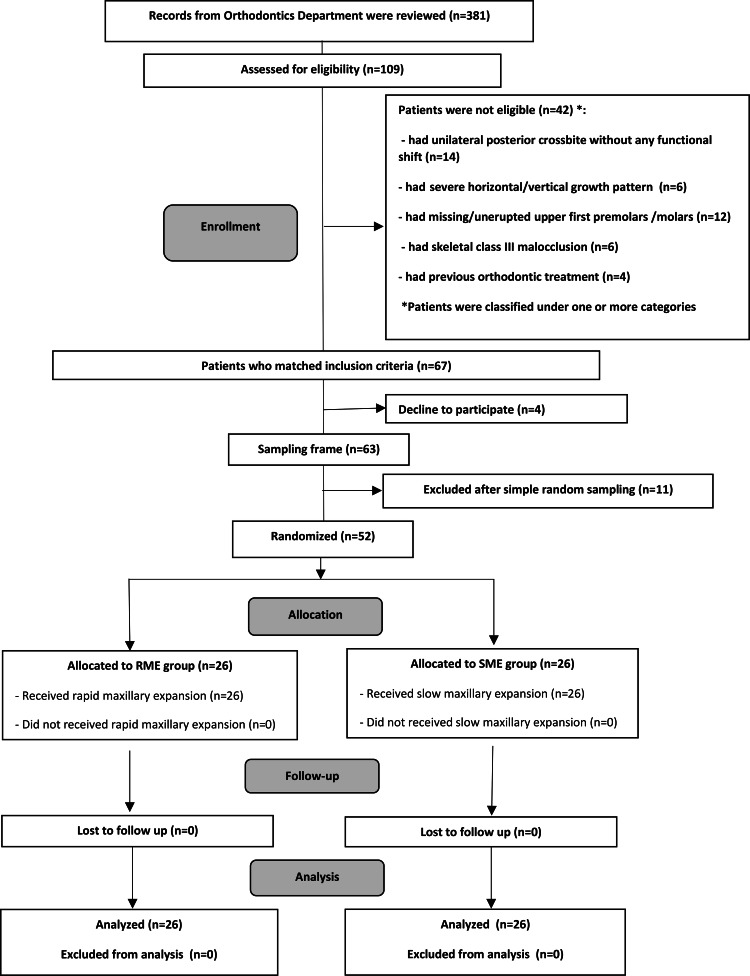
CONSORT flow diagram of patients' recruitment, follow-up, and entry to data analysis.

After examining 109 patients attending the Department of Orthodontics at the University of Damascus, it was found that 67 patients (12-16 years) met the inclusion criteria. The research project was clarified to the patients. Among 63 patients who agreed to join the study, 52 (21 males and 31 females) were randomly selected (Figure 1).

Information sheets were given to all patients, and informed consent forms were collected upon approval. The inclusion criteria were the following: (1) patients in the permanent dentition; (2) chronological age between 12 and 16 years; (3) the presence of a functional unilateral posterior crossbite (with a functional shift) or bilateral posterior crossbite (without any functional shift); (4) skeletal bilateral maxillary constriction (symmetric constriction) was assessed clinically then confirmed radiographically; (5) dental and skeletal class I and II malocclusion; (6) normal and mild vertical growth pattern (Björk's sum between 390 and 406 degrees, Y-axis angle between 63-73 degrees, and SN-MP angle between 26 and 42 degrees); (7) the presence of upper first premolars and molars; (8) no general problems; (9) good oral health; (10) no previous orthodontic treatment. The exclusion criteria involved the following: (1) presence of periodontal diseases; (2) presence of general diseases, syndromes, or cleft lip and palate; (3) patients with previous orthodontic treatment; (4) patients with severe horizontal growth patterns.

Randomization, allocation concealment, and blinding

Patients were allocated between the two groups using a list of random numbers produced by Minitab® software version 17 (Minitab Inc., State College, Pennsylvania, USA) with an allocation ratio of 1:1. The 52 patients were separated randomly into two groups: the RME group (n = 26 patients, mean age ± SD: 13.87 ± 1.31 years; Table 1) and the SME group (n = 26 patients, mean age ± SD: 14.31 ± 1.19 years; Table 1). The allocation sequence was concealed using opaque, locked envelopes. Blinding of the patients and practitioners was not applicable. Therefore, blinding was applied only for data analysis. The first group underwent rapid maxillary expansion, whereas the second group underwent slow maxillary expansion. One of the academic members who was not participatory in this study performed the random allocation sequence and the participants' inclusion into the two groups.

Interventional groups

Rapid Maxillary Expansion Group

**Figure 2:**
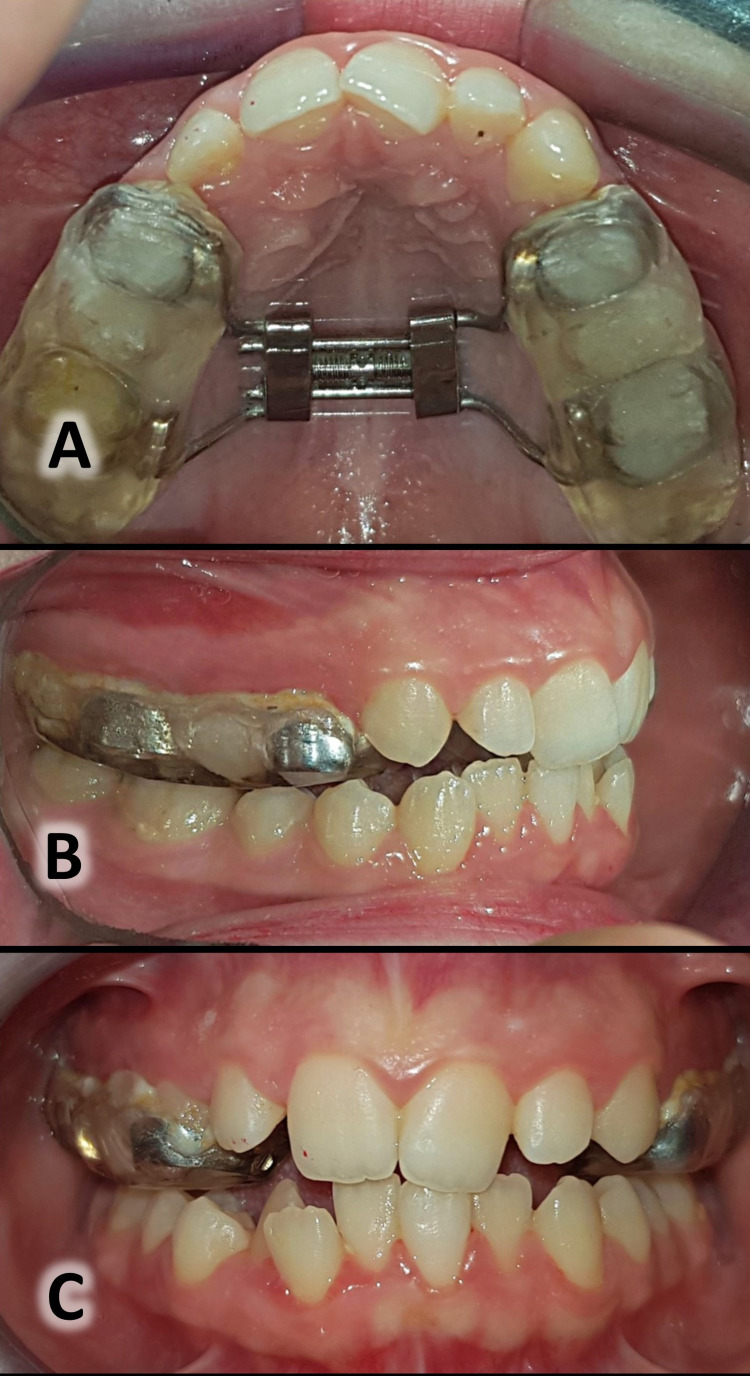
The modified Hyrax-type RME with an acrylic bonded cover (the rapid palatal expander). Occlusal view of the appliance during the rapid maxillary expansion (A); lateral view of the appliance (B); frontal view of the appliance (C).

The expanding appliance used in this study was based on the Hyrax design but with an acrylic cover bonded to the posterior teeth and extending to the occlusal surfaces of them (Figure 2). The patient was asked to activate the expander twice daily (0.4 mm) [22] until gaining a bilateral overcorrection equal to 2-3 mm [23,24]. After that, he/she was asked to stop the expansion and to continue wearing the expander for three months as a retention period [25].

Slow Maxillary Expansion Group

**Figure 3:**
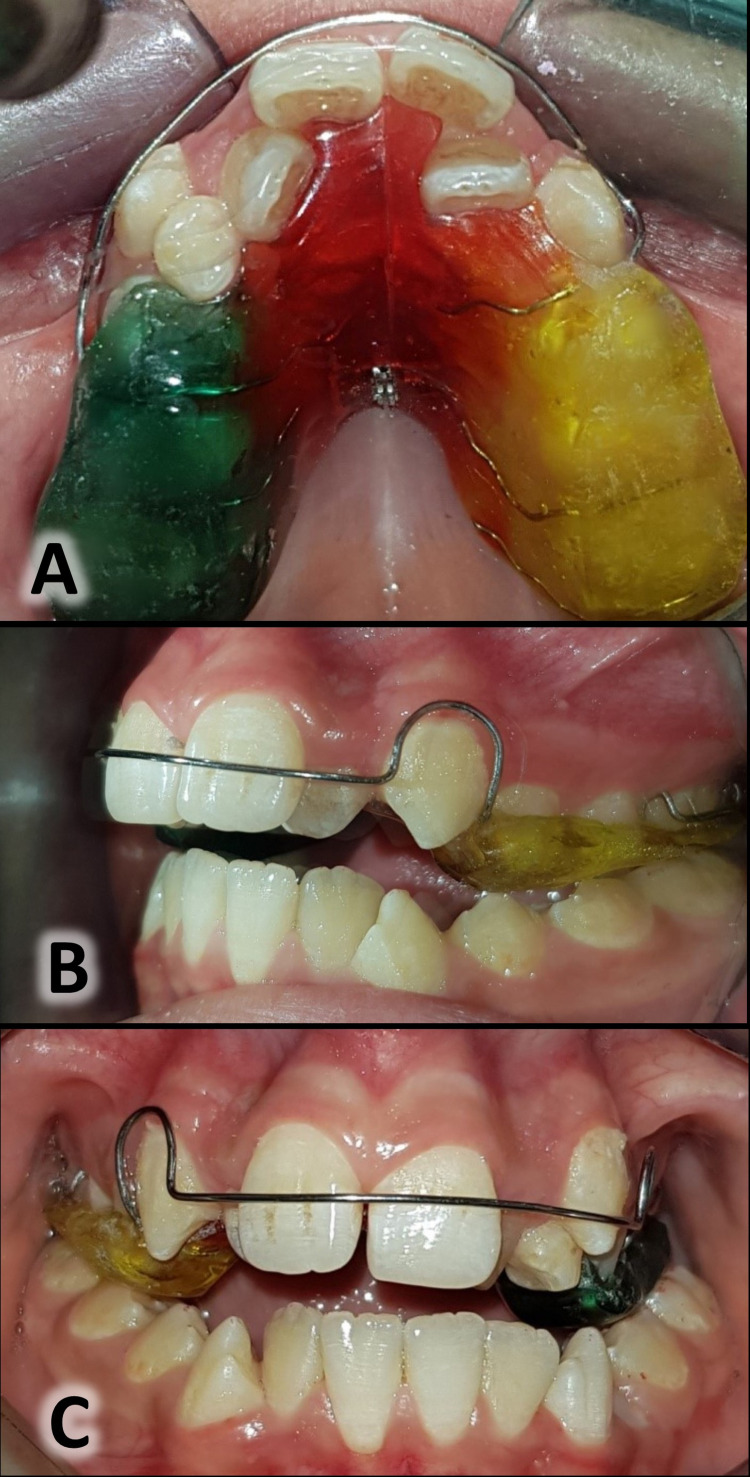
The removable palatal expansion appliance (the slow palatal expander). Occlusal view of the appliance before starting the slow maxillary expansion (A); lateral view of the appliance (B); frontal view of the appliance (C).

The expander used in this study was the removable palatal expansion appliance with posterior bite planes and midline expansion screws (Figure 3). The patients were instructed to wear the appliances 24 hours/day, removing them only for eating or tooth brushing [26]. The patients were asked to activate the expander twice weekly [22] until gaining a bilateral overcorrection equal to 2-3 mm [23,24]. After that, they were asked to stop the expansion and to continue wearing the expander for one month as a retention period [3]. Patients’ compliance was enhanced by verbal motivation at each session, in addition to the repeated telephone calls with patients' parents reminding them of the need for full-time wear.

Outcome measures: pain, discomfort, and functional impairments

**Figure 4:**
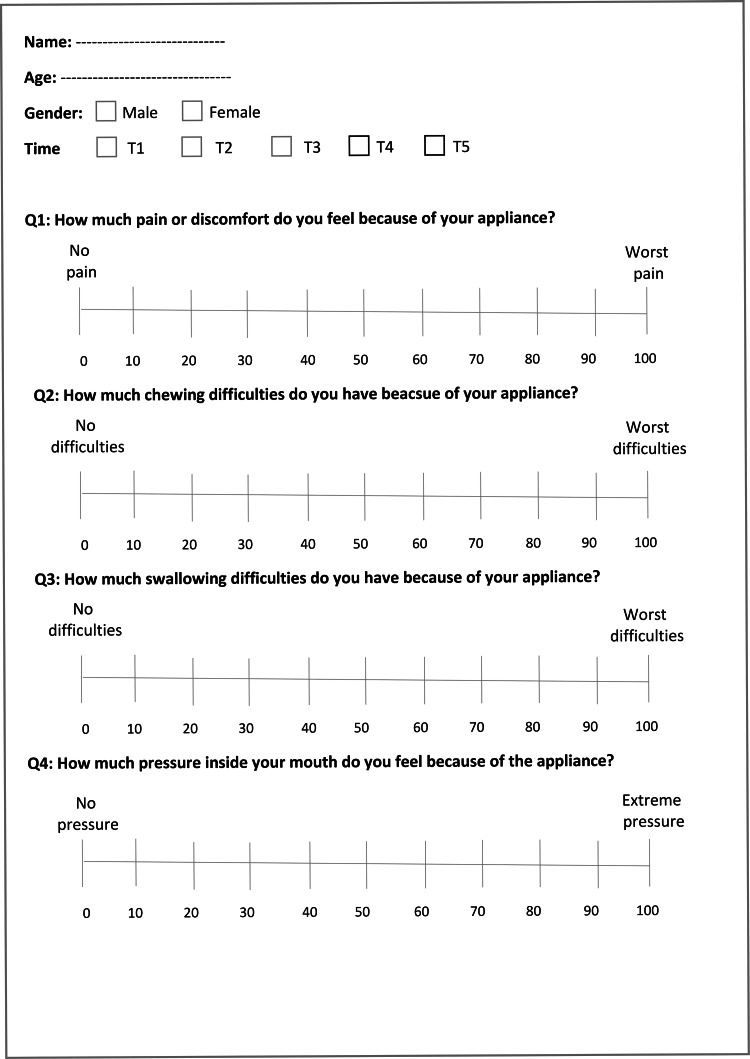
The questionnaire used in the current study.

The outcome measures included the levels of pain, discomfort, and functional impairments within four months after the beginning of the expansion using the attached questionnaire (Figure 4). The questionnaire used in the current study was similar to other questionnaires used for the same purposes in other previous studies [7,11,13,27]. The questionnaire consisted of four questions: (i) the levels of pain and discomfort that patients experienced because of the applied appliance; (ii) the levels of chewing difficulties that patients experienced because of the applied appliance; (iii) the levels of swallowing difficulties that patients experienced because of the applied appliance; and (iv) the levels of pressure inside the mouth that patients experienced because of the applied appliance. The questionnaires were given to patients at five assessment times: 24 hours following appliance insertion (T1), after 7 days (T2), after 15 days (T3), after one month (T4), and after four months (T5). All patients were asked to determine the levels of pain and discomfort and functional impairments they encountered using a visual analog scale (VAS). This scale was a line of 100-mm length from the left side representing no pain and discomfort (i.e., score = 0) to the right side, representing the highest level of pain and discomfort (i.e., score = 100; Figure 4). Patients were instructed to place a mark on the scale (at any point on the drawn line) expressing the degree of their perceived feelings. The level of their response was calculated by measuring the distance from the start point of the line to the specified mark. Patients' inquiries were answered, and any ambiguous questions were clarified by the principal investigator without affecting patients' responses to the questionnaire. If the patient required an analgesic, he/she was asked to fill in the questionnaire first, and then a tablet of paracetamol 500 mg was given. Patients were asked to record the number of pills taken.

Statistical analysis

Minitab® version 17 (Minitab Inc., State College, Pennsylvania, USA) was used for data analysis. The normality of the distribution for each variable was investigated using the Anderson-Darling normality test. Parametric tests were employed when data were normally distributed; otherwise, non-parametric tests were applied. The differences between the two groups were detected using the independent samples t-test or Mann-Whitney U test as appropriate.

## Results

Fifty-two patients (21 males and 31 females) were enrolled in this study. The RME group included 26 patients (16 females and 10 males; average age: 13.87±1.31 years), whereas the SME group included 26 patients (15 females and 11 males; average age: 14.31±1.19). Table 1 shows the baseline features of the recruited patients in each group. No patient dropped out of the study, and hence, the data of 52 patients were entered in the analysis stage (Figure 1). The average active phase in the RME and SME groups was 15 (±2.10) days and 7 (±1.12) months, respectively.

**Table 1:** Basic sample characteristics regarding gender and age. ^†^Employing two-sample t-test. *Employing chi-square test. N: number of patients; SD: standard deviation; Min.: minimum; Max.: maximum; RME: rapid maxillary expansion; SME: slow maxillary expansion.

Group	Gender	N (%)	P-value^*^	Mean age (SD)	Min. age	Max. age	P-value^†^
RME (n=26)	Male	10 (38.46%)	0.131	13.87 (1.31)	12.00	16.00	0.504
	Female	16 (61.53%)					
SME (n=26)	Male	11 (42.30%)		14.31 (1.19)	12.00	16.00	
	Female	15 (57.69%)					
All sample (n=52)	Male	21 (40.38%)		14.09 (1.25)	12.00	16.00	
	Female	31 (59.61%)					

Descriptive statistics of the responses to the four questions are shown in Tables 2 and 3. The pain and discomfort levels in the rapid maxillary expansion group were moderate to severe after 24 hours (66.37; T1), then decreased to become moderate after 7 days during the expansion (51.07; T2), and mild to moderate after 15 days (27.46; T3). While the pain and discomfort levels in the slow maxillary expansion group were mild to moderate after 24 hours (32.16; T1), then decreased to become mild at T2 and T3 (16.46, 12.14, respectively; Table 2). These levels were greater in the rapid maxillary expansion group at T1, T2, and T3, and the differences between the two groups were statistically significant (P<0.001; Table 2). After one month (T4) and four months (T5) after the beginning of the expansion, the pain and discomfort levels were mild in both the rapid and slow maxillary expansion groups without any statistically significant differences between the two groups (p = 0.097, 0.338, at T4 and T5, respectively; Table 2).

**Table 2:** Descriptive statistics of patient-centered variables of questions 1 and 2 at five assessment times in the two groups using the Visual Analog Scale and the results of significance test†. ^†^Employing independent samples t-test or Mann-Whitney test. SD: standard deviation, Min: minimum, Max: maximum, CI: confidence interval, T1: 24 hours after the beginning of the expansion, T2: 7 days after, T3: 15 days after, T4: 1 month after, T5: 4 months after the onset of expansion, RME: rapid maxillary expansion, SME: slow maxillary expansion, (NS): there was no statistically significant difference at P > 0.05, (*): there was a statistically significant difference at P < 0.001.

	Group	Mean	SD	Min	Max	95% CI for difference		P-value	Significance
						Lower bound	Upper bound		
Q1: Pain and discomfort									
T1 (24 h)	RME	66.37	12.71	33.00	87.00	61.34	71.40	<0.001	*
	SME	32.16	11.32	12.00	48.00	27.48	36.83		
T2 (7 d)	RME	51.07	12.72	18.00	71.50	46.03	56.10	<0.001	*
	SME	16.46	6.84	4.00	32.50	13.63	19.28		
T3 (15 d)	RME	27.46	12.32	2.50	48.50	22.58	32.33	<0.001	*
	SME	12.14	6.39	1.00	27.50	9.49	14.78		
T4 (1 m)	RME	12.44	5.46	4.50	23.50	10.28	14.60	0.097	(NS)
	SME	9.24	6.25	0.00	25.50	6.65	11.82		
T5 (4 m)	RME	5.92	5.03	0.00	14.50	3.93	7.91	0.338	(NS)
	SME	8.28	6.19	0.00	24.50	5.72	10.83		
Q2: Chewing difficulties									
T1 (24 h)	RME	47.94	10.16	32.50	67.00	43.92	51.96	0.001	*
	SME	23.94	4.74	17.00	33.50	21.98	25.89		
T2 (7 d)	RME	44.90	10.22	28.50	64.00	40.86	48.95	0.001	*
	SME	19.94	4.74	13.00	29.50	17.98	21.89		
T3 (15 d)	RME	39.05	10.00	25.00	58.00	35.09	43.01	<0.001	*
	SME	17.94	4.74	11.00	27.50	15.98	19.89		
T4 (1 m)	RME	19.68	9.71	5.00	38.00	15.84	23.52	0.001	*
	SME	14.94	4.74	8.00	24.50	12.98	16.89		
T5 (4 m)	RME	0.22	0.50	0.00	2.00	0.02	0.42	0.238	(NS)
	SME	1.58	3.79	0.00	16.00	0.01	3.14		

**Table 3:** Descriptive statistics of patient-centered variables of questions 3 and 4 at five assessment times in the two groups using the Visual Analog Scale and the results of significance test†. ^†^Employing independent samples t-test or Mann-Whitney test. SD: standard deviation, Min: minimum, Max: maximum, CI: confidence interval, T1: 24 hours after the beginning of the expansion, T2: 7 days after, T3: 15 days after, T4: 1 month after, T5: 4 months after the onset of expansion, RME: rapid maxillary expansion, SME: slow maxillary expansion, (NS): there was no statistically significant difference at P > 0.05, (*): there was a statistically significant difference at P < 0.001.

	Group	Mean	SD	Min	Max	95% CI for difference		P-value	Significance
						Lower bound	Upper bound		
Q3: Swallowing difficulties									
T1 (24 h)	RME	25.21	12.71	16.25	34.00	22.99	27.42	<0.001	*
	SME	12.69	3.64	8.50	22.00	11.18	14.19		
T2 (7 d)	RME	23.21	12.72	14.25	32.00	20.99	25.42	<0.001	*
	SME	10.69	3.64	6.50	20.00	9.18	12.19		
T3 (15 d)	RME	22.71	12.32	13.75	31.50	20.49	24.92	<0.001	*
	SME	10.19	3.64	6.00	19.50	8.68	11.69		
T4 (1 m)	RME	22.51	5.46	13.55	31.30	20.29	24.72	<0.001	*
	SME	9.90	3.65	5.50	19.30	8.39	11.41		
T5 (4 m)	RME	0.29	5.03	0.00	2.00	0.00	0.58	0.194	(NS)
	SME	0.95	1.94	0.00	8.00	0.14	1.75		
Q4: Pressure on soft tissue									
T1 (24 h)	RME	27.74	7.57	21.00	48.00	27.74	24.74	0.850	(NS)
	SME	24.36	4.93	8.50	22.00	22.32	26.39		
T2 (7 d)	RME	64.22	11.73	19.00	86.00	59.57	68.86	<0.001	*
	SME	21.36	4.93	6.50	20.00	19.32	23.39		
T3 (15 d)	RME	58.33	11.30	16.00	80.00	53.86	62.803	<0.001	*
	SME	18.36	4.93	6.00	19.50	16.32	20.39		
T4 (1 m)	RME	7.70	6.758	0.00	27.00	5.03	10.37	0.208	(NS)
	SME	8.64	4.69	5.50	19.30	6.70	10.57		
T5 (4 m)	RME	0.07	0.26	0.00	1.00	−0.03	0.17	0.188	(NS)
	SME	0.20	0.40	0.00	8.00	0.03	0.36		

The difficulties of chewing in the RME group were moderate in the first two weeks, then reduced to mild to moderate after one month (47.94, 44.90, 39.05, and 19.68, at T1, T2, T3, and T4, respectively; Table 2). While these difficulties in the SME group were mild to moderate in the first week, they were reduced to mild after 15 days and one month (23.94, 19.94, 17.94, and 14.94, at T1, T2, T3, and T4, respectively; Table 3). The difficulties of chewing were greater in the rapid maxillary expansion group at T1, T2, T3, and T4, and the differences between the two groups were statistically significant (P≤0.001; Table 2). After four months (T5), the difficulties in chewing were almost non-existent in the rapid and slow maxillary expansion groups, with no significant difference between the two groups (0.22 ± 0.50, 1.58 ± 3.79, respectively; P=0.238; Table 2).

The difficulties of swallowing at the assessment times in the first month were mild to moderate in the rapid maxillary expansion group (25.21, 23.21, 22.71, and 22.51; at T1, T2, T3, and T4, respectively; Table 3); while they were mild in the slow maxillary expansion group (12.69, 10.69, 10.19, and 9.90, at T1, T2, T3, and T4, respectively; Table 3). These difficulties were greater in the rapid maxillary expansion group, with statistically significant differences between the two groups (P<0.001, at T1, T2, T3, and T4; Table 3). The difficulties in swallowing were almost non-existent in the rapid and slow maxillary expansion groups after 4 months (T5), without any statistically significant difference (0.29 ± 5.03, 0.95 ± 1.94, respectively; P=0.194; Table 3).

The sense of pressure on soft tissue was mild to moderate after 24 hours in the two groups, without any statistically significant difference (27.74 and 24.36; in the RME and SME groups, respectively; P=0.850; Table 3). In the RME group, the sense of pressure increased to become moderate to severe after 7 days (T2), then it decreased slightly to become moderate after 15 days (T3) (64.22, 58.33 at T2 and T3, respectively; Table 3). On the other hand, the sense of pressure in the SME group remained mild to moderate after 7 days (T2), then decreased slightly to become mild after 15 days (T3) (21.36, 18.36 at T2 and T3, respectively; Table 3). The sense of pressure was greater in the rapid maxillary expansion group at T2 and T3, and the differences between the two groups were statistically significant (P<0.001; Table 3). Then the sense of pressure on soft tissue gradually reduced with time until it almost disappeared after four months in both groups, with no statistically significant differences between them at T4 and T5 (P=0.208 and 0.188, respectively; Table 3). Painkiller consumption was greater in the rapid maxillary expansion group, but the difference was not significant between the two groups (p>0.05).

## Discussion

This is the first randomized controlled trial assessing and comparing the levels of pain, discomfort, and various functional impairments between a removable slow maxillary expander and a bonded rapid maxillary expander in the treatment of skeletal maxillary constriction in growing patients between the ages of 12 and 16. Some researchers have reported that maxillary expansion has caused greater pain and discomfort at such ages in comparison with younger children between the ages of nine and twelve [21,28,29].

Pain is a complicated feeling that differs from one person to another, making the objective measurement of this pain not easy. The VAS was employed in this trial because it is considered one of the most common scales that has been used [6,15,17]. VAS has been demonstrated to be an easy, practical, and valid method of evaluating pain. In addition, it could be used to estimate the variations in pain intensity over time [6].

The levels of pain were statistically and clinically greater in the rapid maxillary expansion group in comparison with the slow maxillary expansion group after 24 hours (T1), 7 days (T2), and 15 days (T3) of the beginning of expansion (P<0.001). These levels were more than two times greater in the rapid maxillary expansion group compared to the slow expansion group in those first three assessment times. The greater levels of pain described in the RME group may be attributed to the heavy and rapid forces that may have caused traumatic splitting of the midpalatal suture and strong compression of the periodontal ligaments. In addition, the inflammatory nature of the highly vascular connective tissue, which formed during the expansion of the midpalatal suture, may be considered another factor in pain perception [13].

In contrast, the slow maxillary expansion delivered a light and regular physiologic force until the required expansion was obtained. It was unethical to bond a slow maxillary expander for a long period of time due to the possible negative effects on the hard tissues (e.g., enamel erosion, attrition, and caries) and soft tissues (e.g., ulcerations and gingivitis). The gradual expansion may have lessened the resistance of circum-maxillary tissue and have given the midpalatal suture tissues enough time to adapt [30]. Therefore, the gradual adaptation of the tissue to the expansion procedure and the lower potential amount of hemorrhaging and ripping may be the reason for the lower level of pain perceived in the SME group [30]. Moreover, the ability to remove the slow maxillary expander during eating and teeth brushing may be another reason for feeling less pain and discomfort.

In addition, the difference in pain levels between rapid and slow maxillary expansion groups may be due to the difference in activation protocols. In this trial, the expander was activated twice weekly in the SME group, while it was activated twice daily in the RME group. The activation protocol created intensive forces in a short time in the RME group (about 2-5 kg per quarter-turn) [30], while it produced lighter forces over a more prolonged duration in the SME group (about 450 gm per week) [30]. This difference in the forces applied may have led to a greater pain level in the RME group.

A gradual decrease over time in the pain levels was noticed in both groups. The greatest levels of pain were recorded after 24 hours of the beginning of the expansion, then they decreased throughout the expansion period until they reached their lowest after the fourth month. The results of this trial were in agreement with the results of studies by Needleman et al. and Baldini et al. [18,20]. They reported that the greatest levels of pain in the RME group were during the first six turns (i.e., the first three days), then these levels diminished in the following days [13,15]. As expansion proceeded, the disruption of the midpalatal tissues reduced, causing a reduction in the pain perception over time. This reduction may be due to the fact that the periodontal tissues adapted to the applied forces or the patients became more accustomed to the appliances [30].

The levels of difficulties in chewing were statistically less in the slow maxillary expansion group at most assessment times (T1, T2, T3, and T4), where the p-value was ≤0.001. Differences were clinically significant at T1, T2, and T3, unlike the difference at T4, which was clinically insignificant (i.e., less than 1 cm on the VAS). This finding can be explained by the difference in the amounts of applied forces, which were greater in the rapid maxillary expansion group. These forces affected the periodontal ligaments of the posterior teeth in a short period that may not have allowed the tissues to adapt adequately, causing chewing difficulties. In addition, the pressure on the periodontal ligaments may have caused the release of chemical factors such as bradykinin, histamine, and prostaglandin and mechanical stimuli. These factors may have activated periodontal ligaments' pain receptors and instigated chewing difficulties [31]. 

Swallowing difficulties were greater in the RME group compared to the SME group. This can be attributed to the large mass of the modified Hyrax-type RME with the acrylic cover being permanently bonded during both expansion and retention periods, and the reduced intraoral space affecting the movement of the tongue. Thus, the movement of food mouthfuls may be negatively affected during the second phase of swallowing. These results were consistent with the study of Abed Al Jawad and Alhashimi which showed that swallowing difficulties were moderate to severe in the first week when using a Hyrax-type RME [32].

During the five assessment times, no significant harm was seen in the slow maxillary expansion group. In contrast, patients reported many difficulties and complications in the rapid maxillary expansion group. Patients in the RME group reported numerous problems, such as repetitive headaches, speaking difficulties, and unsightly diastema. For retentive purposes, the bonded expander was kept in its place for three months. Once the expander was removed, severe ulcers and inflammation were found in the palatal mucosa. Two slow maxillary expanders and one rapid maxillary expander were fractured, and they were repaired within 24 hours.

Limitations

There were various limitations to this trial. First, blinding of the researcher was not possible, which could have produced a detection bias. Second, there was no assessment of any potential root resorption of the posterior teeth or long-term side effects after maxillary expansion. Third, evaluating the periodontal indices, such as the width of the gingiva, alveolar bone, probing depth, and attachment level, was not done. Fourth, assessing the pain, discomfort, and functional impairment should be done using different types of expanders at various age groups. In addition, studying age-related differences between younger and older patients regarding dentoalveolar changes after the two types of expansion is another important aspect. Moreover, evaluation of patient-centered outcomes when the treatment is accomplished using different expansion protocols for the same design of expanders is recommended.

Generalizability

The current study results can be generalized to patients in the adolescence period who use the same or a similar design of maxillary expanders within the same inclusion criteria as in the current study.

## Conclusions

Patients treated with the removable slow maxillary expander reported lower levels of pain and discomfort in the first two weeks and fewer chewing and swallowing difficulties in the first month than patients treated with the bonded rapid maxillary expander. The levels of pain, discomfort, chewing, and swallowing difficulties gradually decreased over a follow-up period of about four months in the rapid and slow maxillary expansion groups. The pressure on soft tissues was greater in the rapid maxillary expansion group after 7 and 15 days of the beginning of the expansion. After four months (T5), the levels of pain and discomfort decreased to their lowest levels, as well as the difficulties of chewing and swallowing and the pressure on soft tissue were almost non-existent in both groups. The current findings may suggest that the removable slow maxillary expanders are more comfortable and less painful treatment alternatives for adolescents with skeletal maxillary constriction when compared to rapid maxillary expansion, provided that similar expansion outcomes are achieved.

## References

[1] Bucci R, D'Antò V, Rongo R, Valletta R, Martina R, Michelotti A (2016). Dental and skeletal effects of palatal expansion techniques: a systematic review of the current evidence from systematic reviews and meta-analyses. J Oral Rehabil.

[2] Alsawaf DH, Almaasarani SG, Hajeer MY, Rajeh N (2022). The effectiveness of the early orthodontic correction of functional unilateral posterior crossbite in the mixed dentition period: a systematic review and meta-analysis. Prog Orthod.

[3] Martina R, Cioffi I, Farella M (2012). Transverse changes determined by rapid and slow maxillary expansion--a low-dose CT-based randomized controlled trial. Orthod Craniofac Res.

[4] Alzabibi BA, Burhan AS, Hajeer MY, Nawaya FR (2021). Short-term effects of the orthodontic removable traction appliance in the treatment of skeletal Class III malocclusion: A randomized controlled trial. Dent Med Probl.

[5] Al-Ouf K, Krenkel C, Hajeer MY, Sakka S (2010). Osteogenic uni- or bilateral form of the guided rapid maxillary expansion. J Craniomaxillofac Surg.

[6] Gecgelen M, Aksoy A, Kirdemir P, Doguc DK, Cesur G, Koskan O, Ozorak O (2012). Evaluation of stress and pain during rapid maxillary expansion treatments. J Oral Rehabil.

[7] Majanni AM, Hajeer MY, Khattab TZ, Burhan AS, Alkhouri I (2020). Evaluation of pain, discomfort, and acceptance during the orthodontic treatment of class iii malocclusion using bone-anchored intermaxillary traction versus the removable mandibular retractor: a randomised controlled trial. J Clin Diagn Res.

[8] Sergl HG, Klages U, Zentner A (1998). Pain and discomfort during orthodontic treatment: causative factors and effects on compliance. Am J Orthod Dentofac Orthop.

[9] Daniels AS, Seacat JD, Inglehart MR (2009). Orthodontic treatment motivation and cooperation: a cross-sectional analysis of adolescent patients' and parents' responses. Am J Orthod Dentofacial Orthop.

[10] Almallah MM, Almahdi WH, Hajeer MY (2016). Evaluation of low level laser therapy on pain perception following orthodontic elastomeric separation: a randomized controlled trial. J Clin Diagn Res.

[11] Gibreal O, Hajeer MY, Brad B (2019). Evaluation of the levels of pain and discomfort of piezocision-assisted flapless corticotomy when treating severely crowded lower anterior teeth: a single-center, randomized controlled clinical trial. BMC Oral Health.

[12] Khattab TZ, Farah H, Al-Sabbagh R, Hajeer MY, Haj-Hamed Y (2013). Speech performance and oral impairments with lingual and labial orthodontic appliances in the first stage of fixed treatment. Angle Orthod.

[13] Saleh M, Hajeer MY, Al-Jundi A (2013). Assessment of pain and discomfort during early orthodontic treatment of skeletal Class III malocclusion using the Removable Mandibular Retractor Appliance. Eur J Paediatr Dent.

[14] Idris G, Hajeer MY, Al-Jundi A (2012). Acceptance and discomfort in growing patients during treatment with two functional appliances: a randomised controlled trial. Eur J Paediatr Dent.

[15] Cossellu G, Lanteri V, Lione R, Ugolini A, Gaffuri F, Cozza P, Farronato M (2019). Efficacy of ketoprofen lysine salt and paracetamol/acetaminophen to reduce pain during rapid maxillary expansion: A randomized controlled clinical trial. Int J Paediatr Dent.

[16] Cesur MG, Aksoy A (2018). Evaluation of perceived pain during the first week of rapid maxillary expansion treatment. Meandros Med Dent J.

[17] Feldmann I, Bazargani F (2017). Pain and discomfort during the first week of rapid maxillary expansion (RME) using two different RME appliances: a randomized controlled trial. Angle Orthod.

[18] Baldini A, Nota A, Santariello C, Assi V, Ballanti F, Cozza P (2015). Influence of activation protocol on perceived pain during rapid maxillary expansion. Angle Orthod.

[19] Halicioğlu K, Kiki A, Yavuz I (2012). Subjective symptoms of RME patients treated with three different screw activation protocols: a randomised clinical trial. Aust Orthod J.

[20] Needleman HL, Hoang CD, Allred E, Hertzberg J, Berde C (2000). Reports of pain by children undergoing rapid palatal expansion. Pediatr Dent.

[21] Ugolini A, Cossellu G, Farronato M, Silvestrini-Biavati A, Lanteri V (2020). A multicenter, prospective, randomized trial of pain and discomfort during maxillary expansion: Leaf expander versus hyrax expander. Int J Paediatr Dent.

[22] Proffit WR WR, Sarver DM (2003). Contemporary treatment of dentofacial deformity. https://www.jco-online.com/media/19032/jco_2004-03-134.pdf.

[23] da Silva Filho OG, Boas MC, Capelozza Filho L (1991). Rapid maxillary expansion in the primary and mixed dentitions: a cephalometric evaluation. Am J Orthod Dentofac Orthop.

[24] Pinheiro FH, Garib DG, Janson G, Bombonatti R, de Freitas MR (2014). Longitudinal stability of rapid and slow maxillary expansion. Dental Press J Orthod.

[25] Lin L, Ahn HW, Kim SJ, Moon SC, Kim SH, Nelson G (2015). Tooth-borne vs bone-borne rapid maxillary expanders in late adolescence. Angle Orthod.

[26] Charavet C, Le Gall M, Albert A, Bruwier A, Leroy S (2019). Patient compliance and orthodontic treatment efficacy of Planas functional appliances with TheraMon microsensors. Angle Orthod.

[27] Alfawal AM, Hajeer MY, Ajaj MA, Hamadah O, Brad B, Latifeh Y (2020). Evaluation of patient-centered outcomes associated with the acceleration of canine retraction by using minimally invasive surgical procedures: a randomized clinical controlled trial. Dent Med Probl.

[28] Ghergu Jianu A, Chaqués-Asensi J, Llamas Carreras JM, Perillo L (2019). Nonsurgical maxillary expansion in adults: report on clinical cases using the Hyrax expander. Minerva Stomatol.

[29] Alghamdi MA, Farsi NJ, Hassan AH (2017). Comparison of oral health-related quality of life of patients treated by palatal expanders with patients treated by fixed orthodontic appliances. Patient Prefer Adherence.

[30] Agarwal A, Mathur R (2010). Maxillary expansion. Int J Clin Pediatr Dent.

[31] Trein MP, Mundstock KS, Maciel L, Rachor J, Gameiro GH (2013). Pain, masticatory performance and swallowing threshold in orthodontic patients. Dental Press J Orthod.

[32] Abed Al Jawad FH, Alhashimi NA (2021). Evaluation of self-perceived pain and jaw function impairment in children undergoing slow and rapid maxillary expansion. Angle Orthod.

